# Urban-rural differences in parenting style in China: A protocol for systematic review and meta analysis

**DOI:** 10.1097/MD.0000000000020592

**Published:** 2020-06-05

**Authors:** Junhua Zhang, Yu Zhang, Fang Xu

**Affiliations:** a School of Psychology, Nanjing Normal University, Nanjing; b School of Education, Jiangsu Key Laboratory for Big Data of Psychology and Cognitive Science, Yancheng Teachers University, Yancheng, China.

**Keywords:** meta-analysis, parenting style, rural, systematic review, urban

## Abstract

**Background:**

Existing literature shows several discrepancies in parenting style between urban and rural China, but conclusions are confusing. Therefore, the aim of this meta-analysis is to consider these inconsistencies and explore the influences of several moderator variables.

**Methods:**

Literature search will be conducted on PubMed, OVID, the Web of Knowledge, China National Knowledge Infrastructure, Wan fang and Chongqing VIP database without language/date/type of document restrictions. The Software Comprehensive Meta-Analysis 3.0 will be used to compare all the selected articles. Moreover, study quality was assessed with a checklist.

**Method and analysis:**

Literature search will be conducted on PubMed, OVID, the Web of Knowledge, China National Knowledge Infrastructure, Wan fang and Chongqing VIP database without language/date/type of document restrictions. We will use Comprehensive Meta-Analysis Software to conduct main meta-analysis. The primary outcomes are scores/subscores measured by the Chinese version of Egna Minnen Beträffande Uppfostran, Parental Bonding Instrument, and any other questionnaires including parenting style.

**Ethics and Dissemination:**

Not needed because no data will be collected.

**Trials registration number:**

INPLASY202050010.

## 1 Introduction

As indicative of the overall emotional climate in the home,^[1]^ there is clear evidence that parenting style can and do have a profound and extensive impact on personality, self-concept and social developmental outcomes of offspring due to the enormous asymmetry in power and competence between adults and children.^[2,3]^ Supportive and authoritative parents were negatively associated to bullying and victimization of children.^[4]^ Authoritarian parenting style is a risk factor for cyberviolence.^[5]^ Authoritarian or rejecting parenting styles was related to behavioral and emotional disorders.^[6]^ A meta-analysis proved that negative parenting behavior is related to a moderate increase of risk for becoming a bully/victim and small to moderate effects on victim status at school.^[7]^ More warmth and behavioral control was related to high level of agreeableness, conscientiousness, and openness and lower levels of neuroticism.^[8]^ Meta-analysis also showed that parenting styles are significantly correlated with aggression of children in mainland China.^[9]^

It is realized that unearthing the specific origins of parenting style is a critically important research objective.^[10]^ According to Jay Belsky's process of parenting model, parenting is multiply determined and is influenced by characteristics of the parent, child, and social context.^[11]^ Bronfenbrenner classified the external environment of the family as meso-systems and exosystem. Mesosystems refer to the relationship between 2 or more systems in which family members participate, such as the relationship between family and school or job while exosystems are the contexts we experience vicariously and yet they have a direct impact on us, such as the political and economic characteristics of society, natural and physical environment, etc. Both mesosystems and exosystem have an impact on parent-child interaction in the family.^[12]^ College-educated parents tend to spend more time with children in the absence of the child's siblings than do less-educated parents.^[13]^ Therefore, the socioeconomic status are linked with parenting style.

From ancient time, there have been 2 Chinas: the myriad agricultural communities of the peasantry in the countryside and the more mobile overlay of walled towns and cities peopled by the families of property and position.^[14]^ The household system since the mid-1950s has strengthened the social structure of the urban-rural divide, and led to the great difference between urban and rural residents.^[15]^ In recent years, China has published a series of policies to promote balanced development between urban and rural areas, and has also achieved positive results. Over the past 2 decades, tremendous changes have occurred in urban and rural areas in China and people's educational level has risen dramatically. However, the traditional dual structure of urban and rural areas still exists.^[16]^ There are huge differences in the geographical environment, social conditions, economic level, cultural education, medical and health care between urban and rural areas, which may have a significant effect on parenting styles.

However, due to the differences in research objects, survey tools, sample size and statistical methods, findings have been mixed. A study found that rural parents had more rejection due to the inequality of economic income and parents’ education level.^[14]^ But another study found that urban parents have more emotional warmth and understanding for their children, but at the same time they have more rejections.^[17]^ A third study found that urban parents have more emotional warmth, understanding and rejection than rural parents.^[18]^ Therefore, effects of rurality on parenting style have not been systematically evaluated. Therefore, there are 2 topics in this study:

1. to compare the difference in parenting style between urban and rural areas;
2. to explore the influences of several moderator variables (types of questionnaires, age of subjects, types of region) on the rural-urban difference in parenting style.

## 2 Methods

The recommendations of Preferred Reporting Items for Systematic Reviews and Meta-Analyses (PRISMA) statement will be followed.^[19]^ The protocol of this systematic review is registered in INPLASY.COM (INPLASY202050010).

### 2.1 Search strategy/syntax

The following electronic databases will be searched with no language/date/type of document restrictions: PubMed, OVID, the Web of Knowledge, China National Knowledge Infrastructure, Wan Fang database and Chongqing VIP database. Search terms in PubMed are as follows: (China or Chinese) and (egna minnen av barn doms uppforstran or Egna Minnen Beträffande Uppfostran (EMBU) or parenting style or parental rearing or parenting behavior or parenting pattern or education style or My memories of upbringing) and (urban or city or cities) and (rural or rural urban differences or rural populations or rural area or countryside).

Additional details about the search strategy/syntax, including search terms for each database, will also be listed. In addition, references of included studies will be scanned to find any potential pertinent study detected with the electronic search.

### 2.2 Study inclusion and exclusion criteria

We will only include studies that met the following criteria:

1. cross-sectional or observational study using EMBU, Parental Bonding Instrument (PBI), and any other questionnaires including parenting style;
2. rural and urban group reported mean (M), standard deviation, number (N) and other parameters such as t, p.

The subjects are normal subjects and criminals and patients will be excluded.

### 2.3 Screening and data extraction

The eligibility process will be conducted in 2 separate stages:

1. Two authors will independently screen titles and abstracts of all nonduplicated papers and exclude those not pertinent.
2. The full-text version articles which get over stage 1 will be downloaded and assessed for eligibility by 2 authors, independently.

Discrepancies will be resolved by consensus between the 2 authors and, if needed, a third senior author will act as arbitrator.

Two researchers will perform independently data extraction; any discrepancies will be resolved by consensus between the 2 authors. The following data will be extracted:

1. Study details: study citation, year(s) of study or publication, region (city or province that can be divided into western, central and eastern regions according to the level of economic development);
2. Participants details, including number, gender distribution, mean and range of age, in both groups;
3. Outcomes: Mean, standard deviation of total score and subscores in both groups and other parameters such as t, p.

### 2.4 Outcomes

The primary outcome will be score measured by the Chinese version of EMBU, PBI and any other questionnaires including parenting style. EMBU is a Swedish self-report scale of perceptions about the behavior used by the respondent's parents in bringing them up. The Chinese version of the questionnaire was revised by Yue and comprises 66 items, with 58 in the six sub-scales about the father (F1 emotional warmth and understanding, F2 punishing, F3 over-interference, F4 favoritism, F5 rejection and F6 over-protection) and 57 in the five sub-scales about the mother (M1; emotional warmth and understanding, M2, over-interference/over-protection, M3 rejection, M4 punishing and M5 favoritism). Responses are on a four-point Likert-type scale, from 1 (never) to 4 (always). Because of the 1-child family planning policy adopted by China before 2011, most of the subjects in the study had no brothers or sisters. F4 and M5 are not suitable for the current situation, so no further investigation was made. The Chinese version of PBI included 2 dimensions of care and overprotection.

### 2.5 Risk of quality assessment

Methodological quality of selected articles will be assessed by using the revised checklist by Chen.^[20]^ It consists of 10 items, including study design, sampling method, rural group information, education level/age of child participants, data collection methods, response rate, comparison group information, measurement, statistical procedure, and data sufficiency.

Two authors will independently assess the quality of each selected study and disagreements will be resolved by further discussion.

### 2.6 Statistical analysis

Analyses will be performed with comprehensive meta-analysis. The urban subjects will be considered as the experimental group, while the rural subjects as the control group. Given the inherent heterogeneity of studies, random-effect model will be used.^[21]^ We will also use the I-squared index to assess the heterogeneity of effect sizes.^[22]^ A significant I-squared indicates that the degree of heterogeneity is greater than would be expected by chance. We will use Egger test and funnel plots to estimate publication biases.^[23]^

### 2.7 Meta-regression and subgroup analysis results

Based on the age of subjects (High school and below vs college and beyond), regions, and questionnaires, we will do subgroup meta-analysis. Additionally, we will plan a meta-regression analysis including parenting style score as outcomes and year of publication, region, age group, and rating on the checklist of quality assessment as regressor.

## 3 Strengths and shortcomings

With regard to the strengths, we preregistered the protocol in INPLASY.COM, reducing the risk of reporting bias. In addition, we will endeavor to perform a comprehensive and systematic search in several databases, with no restrictions in terms of language or document type.

However, our results should be considered very cautiously due to a number of methodologically relevant issues in the retained studies. This is why we would urge caution in the interpretation of these results.

## 4 Conclusions

As to parenting style, what are the similarities and differences between urban and rural parenting style in China? More high-quality and representative studies will also need to be considered.

## Author contributions

**Conceptualization:** Junhua Zhang.

**Formal analysis:** Yu Zhang, Fang Xu, Junhua Zhang.

**Funding acquisition:** Junhua Zhang.

**Project administration:** Yu Zhang, Fang Xu.

**Software:** Junhua Zhang.

**Supervision:** Junhua Zhang.

**Writing – review & editing:** Yu Zhang, Fang Xu, Junhua Zhang.
